# The Potential Prognostic Value of Dual-Imaging PET Parameters Based on ^18^F-FDG and ^18^F-OC for Neuroendocrine Neoplasms

**DOI:** 10.1155/2022/6511179

**Published:** 2022-03-17

**Authors:** Jiale Hou, Tingting Long, Yi Yang, Dengming Chen, Shuo Hu

**Affiliations:** ^1^Department of Nuclear Medicine, XiangYa Hospital, Central South University, No. 87 XiangYa Road, Changsha, Hunan Province, China; ^2^Key Laboratory of Biological Nanotechnology, NHC. No. 87 XiangYa Road, Changsha Hunan Province 410013, China; ^3^National Clinical Research Center for Geriatric Disorders (XIANGYA), XiangYa Central South University, Changsha, Hunan 410008, China

## Abstract

**Background:**

To identify parameters based on dual-imaging ^18^F-AlF-NOTA-octreotide (^18^F-OC) and 18-fluorodeoxyglucose (^18^F-FDG) positron emission tomography (PET) for predicting the prognosis of neuroendocrine neoplasms (NENs).

**Materials and Methods:**

Sixty-six patients (age: mean ± standard deviation (SD): 51.8 ± 11.8 years) who underwent both ^18^F-OC and ^18^F-FDG PET/CT imaging were enrolled in our retrospective study. The following PET parameters were measured: the maximum standardized uptake value (SUV_max_) and the volumetric parameters—^18^F-OC SSR-derived tumor volume (TV) and somatostatin receptor expression (SRE, TV multiplied by the mean standardized uptake value (SUV_mean_)) and the ^18^F-FDG-derived multiple tumor volume (MTV) and tumor lesion glycolysis (TLG). The NETPET grade based on dual-imaging PET images was assessed. Progression-free survival (PFS) was set as an endpoint. Univariate and multivariate survival analyses were performed for PET parameters and clinical tumor data.

**Results:**

In the univariate survival analyses of clinical information, PFS was significantly associated with age (>45.5 vs ≤45.5, years, *P* < 0.034) and the presence of bone metastases (*P* = 0.04). Higher values for the ^18^F-FDG and ^18^F-OC volumetric parameters and the NETPET grade were adverse factors for PFS according to the dual-imaging PET parameters. In the multivariate survival analysis, the NETPET grade and SRE were predictors of PFS in NEN patients.

**Conclusion:**

The NETPET grade is a potential noninvasive prognostic biomarker for NENs.

## 1. Introduction

Neuroendocrine neoplasms (NENs) are a rare and heterogeneous group of malignancies arising from neuroendocrine cells. In the past 40 years, the incidence and prevalence of NENs have continued to rise, with a 6.4-fold increase between 1973 and 2012, across all sites, stages, and grades [1]. The course of the disease and survival of patients with neuroendocrine neoplasms vary [2, 3], even when patients have the same tumor stage and grade. If the prognosis can be effectively predicted, the patient's treatment plan may be changed. Therefore, it is of great significance to find effective parameters for predicting prognosis.

Many controversies and uncertainties persist across some studies regarding the value of clinical-related factors (clinical, laboratory, imaging, and treatment-related factors), pathological factors (histology, classification, and grade), and molecular factors in predicting the prognosis of NEN patients [4, 5]. There is a clinical need to identify ideal prognostic/survival factors for NENs.

The role of positron emission tomography (PET)/computed tomography (CT) is widely recognized in evaluating the cell metabolism, receptor expression, stages, and prognosis of NEN patients. First, most NEN cells express somatostatin receptor 2 (SSTR2). As a result, SSTR imaging can provide accurate information on lesion location, tumor burden, and SSTR expression [6–8], especially for well-differentiated NENs. ^18^F-AlF-NOTA-octreotide (^18^F-OC) is used to image tumors expressing the SSTR2 receptor, exhibiting satisfactory biodistribution and dosimetry profiles with a high NEN lesion detection rate [9–11]. Second, the degree of 18-fluorodeoxyglucose (^18^F-FDG) labelling is related to the aggressiveness of NENs and is one of the predictors of tumor progression [12]. In addition, ^18^F-FDG PET is more likely to be positive with increasing histological grade [13]. Therefore, integrating metabolic and receptor imaging may be valuable in providing more beneficial information on the prognosis of patients with NENs [14–16]. Significant ^18^F-FDG-avid and nonavid SSTR lesions might indicate NEN patients with aggressive diseases, active metabolism, and poor prognosis. In contrast, the lack of ^18^F-FDG uptake on detected metastatic NEN lesions might suggest low-grade, metabolically inactive conditions in NEN patients, resulting in an inert course and a better prognosis.

Recently, a visual evaluation method [17] based on dual ^18^F-FDG/^18^F-OC imaging (NETPET grade) has emerged. The NETPET grade is significantly related to the prognosis of patients and is easy to calculate for patient management and further research because it can summarize the information from the dual ^18^F-FDG and SSTR PET imaging through a simple parameter. This grading scheme can reflect the spatial consistency of the lesions, the relative uptake of the two imaging radiotracers by their respective lesions, and the degree of disease. However, the value of the NETPET grade using dual-tracer imaging over common, semiquantitative PET parameters in predicting the prognosis of NEN patients has not been determined.

This study evaluates the prognostic value of dual-imaging PET parameters based on ^18^F-FDG and ^18^F-OC imaging for NENs.

## 2. Materials and Methods

### 2.1. Patients

We conducted this retrospective selected data study at Xiangya Hospital and Ethics Committee approval (No. 20181001) was obtained for this trial, and the requirement for informed consent was waived.

One hundred thirty-eight patients with pathologically confirmed NEN in our hospital between August 2017 and December 2020 were included in this study. The exclusion criteria were as follows: (1) the presence of other malignant tumors (*n* = 2), (2) multiple endocrine neoplasms (*n* = 1), (3) a lack of positive lesions on ^18^F-OC and ^18^F-FDG scans (*n* = 4), (4) insufficient follow-up (*n* = 29), (5) only one scan of ^18^F-OC and ^18^F-FDG PET (*n* = 35), and (6) more than one month between scans was *n* = 1. Finally, the remaining 66 patients were enrolled. When a patient underwent multiple PET scans, the first examination was selected. According to the World Health Organization (2010) classification system, patients with NENs were classified as G1, G2, and G3, with reference to mitosis and Ki-67 index. The mean follow-up period ranged 19-187 weeks (mean ± SD: 52.5 ± 34.8 weeks). Patient characteristics, including age, gender, primary tumor location, sites of metastases, Ki-67 index (%), grade, and treatment, were collected at baseline (Table 1).

### 2.2. Radiopharmaceutical Preparation of ^18^F-OC

Details of the labeling technique and quality control of ^18^F-OC are described in detail in a previous publication [18]. In short, firstly, ^18^F-fluoride was absorbed on a Sep-Pak QMA-carbonate Light cartridge (Waters, Massachusetts) and then eluted into a reaction vessel with 300 *μ*L of saline. Second, the reaction solution was mixed with NOTA-octreotide (300 *μ*g) in anhydrous acetonitrile (1 mL) and acetamidophos (500 *μ*L, pH 4.0), stirred for 3 minutes and heated to 100°C. Ten minutes later, the radiolabeled peptide was purified using high-performance liquid chromatography (HPLC). The eluted product fraction was collected at the retention time corresponding to ^18^F-OC, diluted with 50 mL of H_2_O, and passed through a C18 cartridge (Plus Sep-Pak; Waters) to remove the acetonitrile and trifluoroacetic acid. Next, ^18^F-OC was eluted into a collection bottle with 2 mL of ethanol and diluted with 15 mL of saline. Finally, the solution was filtered through a 0.2 *μ*m sterile filter (Millex GV, Sterile, 0.22 mm; Millipore, Darmstadt, Germany) into a sterile vacuum flask. The final chemical and radiochemical purity of the product was determined by HPLC. The radiochemical purity was greater than 99.0%, and no impurities were found.

### 2.3. PET/CT Protocol

The PET/CT images were acquired on a General Electric Discovery PET/CT 690 Elite scanner (General Electric Health care, Waukesha, WI). The scanning interval between the ^18^F-FDG and ^18^F-OC scans was less than 1 month but more than one day. For the ^18^F-FDG scan, the patients fasted for at least 6 hours before the scan, and the serum glucose level needed to be less than 180 mg/dl. Both ^18^F-FDG and ^18^F-OC scans were performed using PET approximately 1 hour after intravenous (IV) injection of the radiotracer at a dose of 3.7 to 4.44 MBq (0.1–0.12 mCi) per kilogram of body weight. Whole-body (top of the skull to midthigh) low-dose CT scans (120 kV; automatic mAs; pitch, 1; slice thickness, 3.75 mm; matrix, 512 × 512) were performed. After a CT, the PET scan was performed on the same anatomical area immediately. Each bed position was held for 2 minutes for each patient. The PET datasets were reconstructed with a 3-dimensional ordered-subset expectation maximization (OSEM) algorithm with 2 iterations and 23 subsets.

### 2.4. Progression-Free Survival (PFS)

PFS was calculated as the time interval from the start of the first PET/CT scan to disease progression or tumor-related death. If no progression or tumor-related death occurred within the follow-up period, the patient was censored at the date of the last available diagnostic imaging or comprehensive clinical assessment. According to the RECIST 1.1 standard [19], tumor progression was defined as a significant increase in tumor size or the appearance of new metastatic lesions.

### 2.5. Image Analysis

PET/CT images were evaluated using VCAR software in AW workstation 4.6 (General Electric Healthcare). Radiotracer uptake higher than the background that could not be explained by physiological uptake was considered to indicate a positive lesions. The PET/CT images were reviewed by an experienced nuclear medicine physician.

### 2.6. Semiquantitative Parameters Analysis

The software provides an automatic method of delineating the volume of interest (VOI) based on the threshold SUV, with a chosen cut-off of 50%. Manual microadjustment was used to avoid the presence of surrounding physiological uptake and adjacent lesions. A VOI measuring less than 0.1cm^3^ was excluded. The software enables automatic generation of the maximum standardized uptake value (SUV_max_), volume, and other information in individual VOIs. To minimize the overestimation of volumetric parameters, overlapping between adjacent VOIs was strictly avoided.

The following semiquantitative PET parameters were assessed: SUV_max_ for both ^18^F-OC and ^18^F-FDG PET, SSR-derived tumor volume (TV), somatostatin receptor expression (SRE) for the ^18^F-OC PET scan, and metabolic tumor volume (MTV) and tumor lesion glycolysis (TLG) for the ^18^F-FDG PET scan. The SRE was obtained by multiplying the SSR-derived TV with the mean SUV within the same VOI.

The lesion SUV_max_ with the highest ^18^F-OC and ^18^F-FDG uptake in each patient was subjected to statistical analysis. We also calculated and used the sum of the SSR-derived TV, SRE, MTV, and TLG in all detected lesions per patient in this analysis.

### 2.7. NETPET Grade Based on Dual-Tracers Analysis

To determine the NETPET grade, we referred to the evaluation method published by Chan et al. [17]. Briefly, the adopted strategy was to determine the single lesion with the highest ^18^F-FDG uptake relative to its ^18^F-OC uptake after excluding other non-NEN reasons for the ^18^F-FDG increase with the SUV_max_ thresholds set at 7.0 for ^18^F-FDG and 15.0 for ^18^F-OC, as these are the values reported for use in clinical practice.

**Figure 1:**
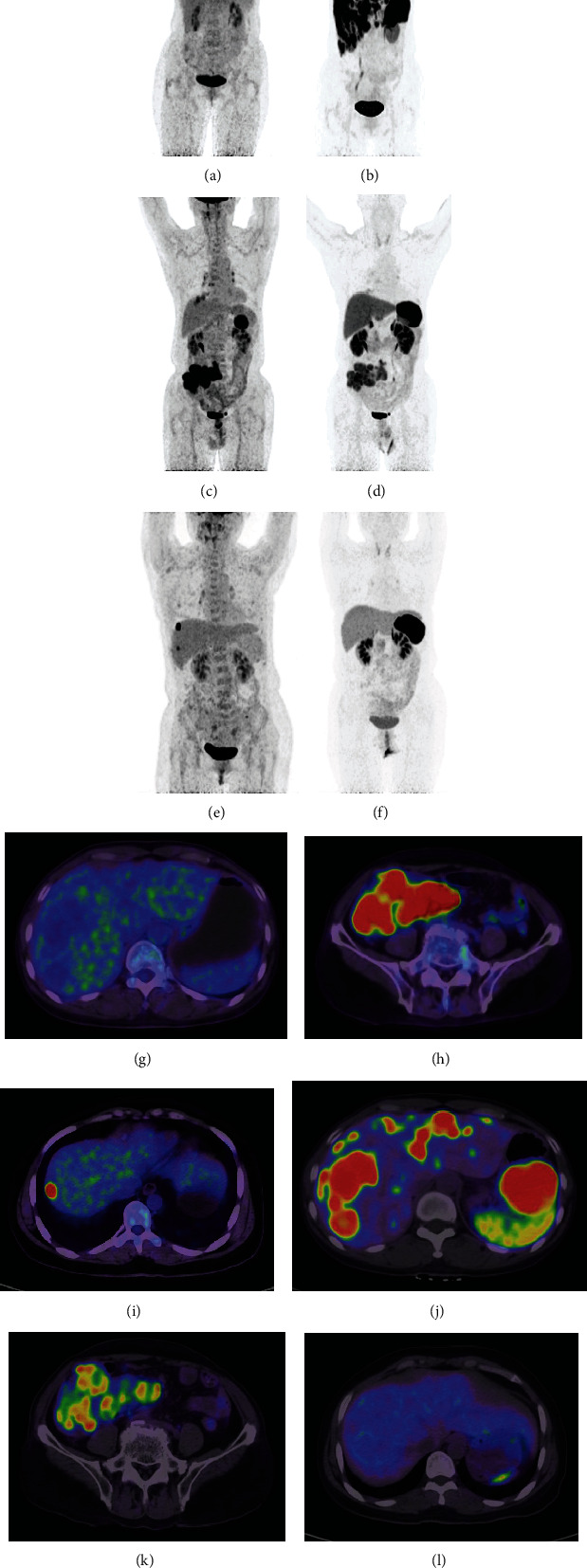
Shows the performance of ^18^F-FDG and ^18^F-OC in three patients. The maximum intensity projection (MIP, a–f) images from the respective PET datasets are shown. Images a, b, g, and j are from a patient with a G1 neuroendocrine tumor that had a NETPET score of P1, indicating that the scans are negative for ^18^F-FDG (a and g) and positive for ^18^F-OC (b and j) in the liver and abdominal cavity. Images c, d, h, and k are from a patient with a G3 neuroendocrine tumor of the ileocecum and lymph nodes and show that the lesions have greater avidity for ^18^F-FDG (c and h) than ^18^F-OC (d and k), with a NETPET score of P4b. Images e, f, i, and l are from a patient after rectal G3 neuroendocrine tumor surgery that had a NETPET score of P5, indicating that the scans are negative for ^18^F-OC (f and l) and positive for ^18^F-FDG (e and i) in the liver.

The NETPET grade was classified on a P0-5 scale based on visual assessment. A NETPET grade of P0 represented a regular scan on both ^18^F-OC and ^18^F-FDG PET. A grade of P1 was given if there was only ^18^F-OC-avid disease, no ^18^F-FDG uptake in any lesions (Figure 1). If the opposite was observed, the grade was P5, and. P2-4 indicated lesions with both noticeable ^18^F-FDG uptake and ^18^F-OC uptake. More specifically, P2 indicated weaker ^18^F-FDG uptake than ^18^F-OC uptake. The lesions with ^18^F-FDG uptake equal to ^18^F-OC uptake were graded as P3. When ^18^F-FDG uptake was higher than ^18^F-OC uptake, the grade was classified as P4. One particular situation, in which a lesion had the most elevated ^18^F-FDG uptake but no ^18^F-OC uptake and another had one second-highest ^18^F-FDG uptake with positive (but less) ^18^F-OC uptake was also graded P4 (Figure 1). If there were more than 2 such similar lesions, it was defined as P5 (Figure 1). Each P2-4 group was classified into subgroup a with at most two described lesions and subgroup b with more than two lesions.

### 2.8. Statistics

Continuous variables are reported as the mean ± standard deviation (SD) or median (interquartile range (IQR). According to the cutoff value generated from the receiver operating characteristic (ROC) curve, the PET parameters were divided into two groups (low and high). The survival curve was established by the Kaplan-Meier method and analyzed by the log-rank test. Univariate and multivariate analyses were performed on the prognostic variables of PFS using Cox regression analysis (Forward: LR used in multivariate analysis), and the results are expressed as hazard ratios (HRs) and 95% confidence intervals (CIs). Spearman's correlation coefficient was used to evaluate histological grade and NETPET grade. Statistical analysis was performed using Graphpad Prism (version 8.0 for windows, Graphpad Software) and SPSS software version 24 (SPSS Inc., IBM, Chicago, USA). *P* < 0.05 was considered statistically significant.

## 3. Results

### 3.1. Patient Characteristics

A total of 66 NEN patients (46 men and 20 women; mean age: 51.8 ± 11.8 year (y), range from 17-73 y) were enrolled in our study. NENs of pancreatic origin were found in 34.8% of patients, those gastrointestinal origin in 37.9%, those lung origin in 4.6%, and those originating from other sites in the remaining 22.7%. According to the 2010 WHO grade, 14 patients had grade 1 (21.2%), 30 patients had grade 2 (45.5%), and 22 patients had grade 3 (33.3%). Metastatic sites included the liver (*n* = 41 and 62.1%), bone (*n* = 17 and 25.8%), lymph nodes (*n* = 40 and 60.6%), and lung (*n* = 9 and13.6%). The characteristics of the 66 patients are summarized in Table 1.

### 3.2. ^18^F-OC and ^18^F-FDG PET/CT Variables

Among ^18^F-OC PET/CT parameters, the median SSTR-derived TV was 38.16 ml (range: 0-1278.86), the median SRE was 727.94 (0-52667.68), and the median SUV_max_ was 56.79 (0-365.95). Among the ^18^F-FDG PET/CT parameters, the median MTV was 14.72 ml (range: 0-956.23) and the median TLG was 143.85 (0-21226.01). The median SUV_max_ was 18.55 (0-151.08) (shown in Table 1). In our study, the patients were divided into P1-5 according to NETPET grade (Table 2). P1 represented solely ^18^F-OC-positive, P2-4 were ^18^F-OC-positive/^18^F-FDG-positive disease, and P5 represented ^18^F-OC -negative/^18^F-FDG-positive.

### 3.3. Evaluation of Clinicopathological Features and Dual-Imaging PET Parameters for the Prognosis of NEN Patients

PFS was confirmed during the follow-up period in 45 patients (68.2%) according to the RECIST 1.1 criteria and comprehensive clinical assessment, with a median of 52 weeks (IQR:40.5-66.5 weeks). Regarding clinical information, patients aged >45.5 years showed a worse prognosis in the univariate analysis (*P* = 0.034). However, the subgroups for gender, Ki-67 (>5%), histological grade, CgA, CEA, and NSE positivity showed no significant differences in PFS. Regarding metastatic site, patients with bone metastases were significantly associated with a shorter PFS (*P* = 0.04). However, there were no significant differences for other metastases sites, such as liver, lung, and lymph node metastases.

Among the ^18^F-OC PET parameters, a higher SRE (>20300.3) predicted a poor outcome (HR: 2.403, 95% CI: 0.703-8.208, *P* = 0.034). Among the ^18^F-FDG PET parameters, higher values of the volumetric parameters MTV (>1.15 ml) and TLG (>45.54) were significantly associated with a shorter PFS (*P* = 0.025 and 0.026, respectively, Table 3). However, the SSR-derived TV, SUV_max_ based on ^18^F-OC and ^18^F-FDG PET, and liver, lymph node, and lung metastases did not show a significant difference in PFS between the two groups (all *P* > 0.05, Table 3).

**Figure 2:**
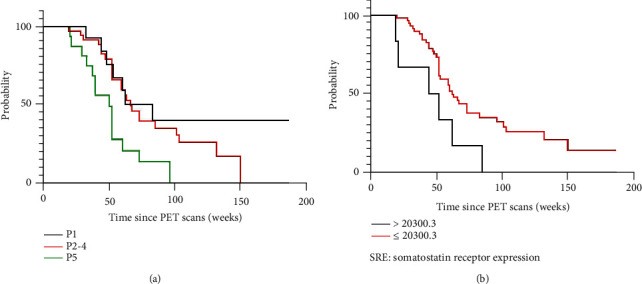
Kaplan-Meier curves for the NETPET grade (a) and SRE (b).

On multivariate analysis (Table 3), significant differences in PFS were observed for the NETPET grade and SRE. A high NETPET grade and higher SRE (>20300.3) were associated with poor survival (Figure 2, *P* = 0.011 and 0.039). As a result, they were regarded as independent predictors for poorer PFS.

The NETPET grade was also significantly also associated with WHO 2010 histological grade (Spearman's test for correlation, *r* = 0.543, *p* < 0.0001).

## 4. Discussion

This project mainly studied the value of various parameters of dual-tracer imaging radiotracers (^18^F-FDG and ^18^F-OC) in the prognosis of patients with NENs. Herein, some critical parameters were proven to have a potential predictive value in NEN patient management.

The complementary adoption of SSTR-PET and ^18^F-FDG PET tracers may be valuable in the diagnostic workup of NETs, which has been described previously [20, 21]. SUV_max_ has been widely studied in predicting the prognosis of NENs, and it is a common semiquantitative indicator in PET imaging. However, the SUV_max_ of ^18^F-FDG and ^18^F-OC PET in our study did not predict PFS in patients with NENs. A possible explanation is that SUV_max_ only represents the largest pixel in a tumor, rather than the overall tumor, and is affected by many factors, such as tumor size and noise [22]. In contrast, the whole tumor volume based on PET can reflect the condition of the entire tumor, and it is likely to be the more accurate parameter for reflecting prognosis. In our study, volumetric parameters based on ^18^F-OC and ^18^F-FDG were essential factors for predicting prognosis in NEN patients. The expression of SSTR2 in whole tumors can reflect the tumor burden. A previous study showed that the expression of SSTR is an independent favorable prognostic factor for NEN patient survival [23]. Tumor uptake on SSTR2 imaging is correlated with SSTR2 expression on immunohistochemistry [24]. In addition, SSTR2 imaging has higher accuracy in predicting patient prognosis than SSTR2 immunohistochemistry. We consider that SRE reflects the SSTR burden of the NEN patient's whole body, while SSTR2-immunohistochemistry can only reflect the expression of a certain lesion due to sampling error. Therefore, the SRE based on SSTR imaging may be more accurate in determining the individual prognosis. Additionally, a higher MTV and TLG based on ^18^F-FDG PET are related to a poorer PFS, which reflects the metabolic characteristics of the whole tumor. The importance of volume parameters based on ^18^F-FDG in prognosis has been extensively researched in other types of cancer [25–27].

The presence of bone metastases was also an indicator of poor prognosis in univariate analysis. Bone metastases usually occur in advanced NENs and are inversely associated with prognosis [28]. Liver metastases are one of the most common sites of NENs. Liver metastases are the strongest predictor of survival in NEN patients regardless of the primary site [29]. The 5-year survival rate of NEN patients with liver metastases is significantly lower than that of patients without liver metastases (13-54% vs75-99%) [30]. However, we do not find that the existence of liver metastases was related to PFS in our study. One possible reason is that the proportion of liver metastases was significant, and there was heterogeneity among the enrolled patients in our group.

In our study, the NETPET grade based on dual-imaging PET was an independent predictor of the prognosis of NENs. Previous studies have shown that integrating SSTR and ^18^F-FDG PET information can produce a comprehensive biomarker [31–33]. However, it can be complicated to describe the two PET scans, which represent different affinity patterns, in a pure text report. In addition, it may be difficult for the referring physician to extract a summary of relevant findings, especially in a plain text report. Therefore, the NETPET scoring system, based on visual assessments, was first reported by Chan et al. in 2017 [17] to simplify the complexity of dual-imaging PET parameter evaluation and make it easier for readers to observe and analyze. In addition, we noticed that the NETPET grade was significantly correlated with histological grade. However, the histological grade was not associated with PFS despite its recognized prognostic factor [34], which is consistent with the study of Chan et al. [17]. We think that this may be because the PET scans provide a total body evaluation, although the number of cases enrolled in this study is small.

In this study, univariate analysis suggested that the volumetric parameters MTV and TLG based on ^18^F-FDG-PET, the SRE based on ^18^F-OC PET, and the NETPET grade predicted PFS. In multivariate analysis, only the NETPET grade and SRE were found to be independent predictors of PFS. The NETPET can be judged by visual assessment, making it easier for other physicians to read. The PET volume parameters need to be evaluated for lesions shown in the whole body. Although some software can perform this evaluation, for smaller lesions, the limited spatial resolution of PET, and the partial volume effect may result in a negative evaluation. In addition, while using ^18^F-FDG or SSTR alone, the sensitivity and specificity of the resolution of PET for different lesion pathological grades are different [35]. Dual-imaging provides relevant information about tumor behavior and aggressiveness and therefore is conducive to the development of more personalized treatment strategies and solves the limitations related to histopathological grading and tumor heterogeneity. Therefore, NETPET has the potential to be a prognostic factor for NENs, and but larger cohort is needed for prospective verification in the future.

### 4.1. Limitation

Some limitations should be acknowledged. First, our study was conducted in only one hospital without external validation. The reconstruction algorithm and other information need to be clarified before the findings can be promoted. Second, we did not study overall survival because some patients did not have sufficiently long-term follow-up data. Additionally, our study did not consider the influence of different treatment options before and after the examination, which may have had a certain impact on the results. Finally, since this study is retrospective in nature, there may be selection bias. In the future, we still need to perform a case-by-case analysis before clinical use.

## 5. Conclusion

Our data consider that volume parameters based on ^18^F-FDG and ^18^F-OC PET are related to the prognosis of patients with NENs. Besides, the NETPET grade also can be used as one of the independent predictors of the prognosis of NEN patients. Although these results need to be validated in subsequent cohorts, dual-imaging have an impact on prognostic evaluation of NENs and the intensity of surveillance strategies.

## Figures and Tables

**Table 1:** Patient characteristics.

Characteristic	Value
Total number of patients	66
Age (year)	51.8 ± 11.8
Gender	Male:46, female: 20
Primary tumor site (*n* %)	
Pancreas	23 (34.8%)
Gastrointestinal	25 (37.9%)
Bronchopulmonary	3 (4.6%)
Others^a^	15 (22.7%)
WHO 2010 Grade (*n* %)	
G1	14 (21.2%)
G2	30 (45.5%)
G3	22 (33.3%)
Time since diagnosis (weeks)	0-346
Treatment	
Before PET/CT	
Surgical	22 (33.3%)
33Medical^b^	18 (27.3%)
Liver-directed treatment^c^	2 (3.0%)
Radiation	2 (3.0%)
No treatment	30 (45.5%)
After PET/CT	
Surgical	11 (16.7%)
Medical^b^	48 (72.7%)
Liver-directed treatment^c^	11 (16.7%)
Radiation	5 (7.6%)
No treatment	7 (10.6%)
^18^F-OC PET parameters, median (range)	
SUV_max_	56.79 (0-365.95)
SSTR-derived TV	38.16 (0-1278.86)
SRE	727.94 (0-52667.68)
^18^F-FDG PET parameters, median (range)	
SUV_max_	18.55 (0-151.08)
MTV	14.72 (0-956.23)
TLG	143.85 (0-21226.01)
Presence of metastases (*n* %)	
Liver metastases	41 (62.1%)
Bone metastases	17 (25.8%)
Lymph node metastases	40 (60.6%)
Lung metastases	9 (13.6%)
CgA (positive, *n* %)	55 (83.3%)
CEA (positive, *n* %)	8 (12.1%)
NSE (positive, *n* %)	8 (12.1%)
Tumor progression	
Yes	45 (68.2%)
No	21 (31.8%)

^a^Others included six patients with a mediastinum lesion origin, six patients with unknown primary sites, one patient with an origin in the throat, one patient with an origin in the breast, and one patient with an origin in the biliary tract. ^b^Medical treatments included cold somatostatin analog and other anticancer drugs. ^c^Liver-directed treatments included transcatheter arterial chemo- or radioembolization and microwave ablation for liver metastases. SSTR-derived TV: somatostatin receptor-derived tumor volume; SRE: somatostatin receptor expression; MTV: metabolic tumor volume; TLG: tumor lesion glycolysis; CgA: chromograninA; CEA: carcinoembryonic antigen; NSE: neuron-specific enolase; PFS: progression-free survival; IQR: interquartile range; PRRT: peptide receptor radionuclide therapy.

**Table 2:** Retrospective classification of included patients by NETPET grade according to the study of Chan et al.

NETPET grade	Number
P1	14
P2a	6
P2b	10
P3a	5
P3b	1
P4a	5
P4b	10
P5	15

**Table 3:** Risk factors from univariate analysis and multivariate analysis in predicting PFS.

Variables	Univariate analysis			Multivariate analysis		
	HR	95% CI	*P*	HR	95% CI	*P*
Age (≤45.5 vs >45.5, years)	0.5256	0.256-1.080	0.034^∗^	/	/	0.070
Gender (male vs female)	1.181	0.632-2.209	0.602	/	/	/
Time since diagnosis	1.768	0.851-3.674	0.127			
WHO 2010 grade	1.386	0.939-2.047	0.101	/	/	/
Sites of metastases						
Liver metastases	0.560	0.297-1.058	0.074	/	/	/
Lymph node metastases	0.822	0.449-1.506	0.526	/	/	/
Bone metastases	0.524	0.282-0.972	0.04^∗^	/	/	0.452
Lung metastases	1.003	0.423-2.376	0.995	/	/	/
^18^F-OC parameters						
SUV_max_ (≤163.18 vs >163.18)	0.884	0.492-1.590	0.675	/	/	/
SSTR-derived TV (≤174.16 mL vs >174.16 mL)	1.666	0.833-3.333	0.095	/	/	/
SRE (≤20300.3 vs >20300.3)	2.403	0.703-8.208	0.034^∗^	2.511	1.047-6.022	0.039^∗^
^18^F-FDG parameters						
SUV_max_ (≤38.93 vs >38.93)	1.268	0.641-2.507	0.456	/	/	/
MTV (≤1.15 mL vs >1.15 mL)	2.703	1.130-6.466	0.025^∗^	/	/	0.506
TLG (≤45.54 vs >45.54)	2.127	1.164-3.885	0.026^∗^	/	/	0.433
NETPET grade	1.849	1.144-2.990	0.012^∗^	1.917	1.159-3.170	0.011^∗^
CgA (+ vs -)	0.890	0.427-1.854	0.756	/	/	/
CEA (+ vs -)	0.534	0.234-1.216	0.135	/	/	/
NSE (+ vs -)	0.862	0.361-2.058	0.738	/	/	/

SSTR-derived TV: somatostatin receptor-derived tumor volume; SRE: somatostatin receptor expression; MTV: metabolic tumor volume; TLG: total lesion glycolysis; CgA: ChromograninA; CEA: Carcinoembryonic antigen; NSE: Neuron-specific enolase.

## Data Availability

The image data used to support the findings of this study are available from the corresponding author upon request.

## References

[1] Dasari A., Shen C., Halperin D. (2017). Trends in the incidence, prevalence, and survival outcomes in patients with neuroendocrine tumors in the United States. *JAMA Oncology*.

[2] Hallet J., Law C. H., Cukier M., Saskin R., Liu N., Singh S. (2015). Exploring the rising incidence of neuroendocrine tumors: a population-based analysis of epidemiology, metastatic presentation, and outcomes. *Cancer*.

[3] Zandee W. T., de Herder W. W. (2018). The evolution of neuroendocrine tumor treatment reflected by ENETS guidelines. *Neuroendocrinology*.

[4] Lee L., Ito T., Jensen R. T. (2019). Prognostic and predictive factors on overall survival and surgical outcomes in pancreatic neuroendocrine tumors: recent advances and controversies. *Expert Review of Anticancer Therapy*.

[5] Chan D. L., Clarke S. J., Diakos C. I. (2017). Prognostic and predictive biomarkers in neuroendocrine tumours. *Critical Reviews in Oncology/Hematology*.

[6] Binderup T., Knigge U., Mellon Mogensen A., Palnaes Hansen C., Kjaer A. (2008). Quantitative gene expression of somatostatin receptors and noradrenaline transporter underlying scintigraphic results in patients with neuroendocrine tumors. *Neuroendocrinology*.

[7] Bozkurt M. F., Virgolini I., Balogova S. (2017). Guideline for PET/CT imaging of neuroendocrine neoplasms with 68Ga-DOTA-conjugated somatostatin receptor targeting peptides and 18F–DOPA. *European Journal of Nuclear Medicine and Molecular Imaging*.

[8] Barrio M., Czernin J., Fanti S. (2017). The impact of somatostatin receptor-directed PET/CT on the management of patients with neuroendocrine tumor: a systematic review and meta-analysis. *Journal of Nuclear Medicine*.

[9] Long T., Yang N., Zhou M. (2019). Clinical application of 18F-AlF-NOTA-octreotide PET/CT in combination with 18F-FDG PET/CT for imaging neuroendocrine neoplasms. *Clinical Nuclear Medicine*.

[10] Pauwels E., Cleeren F., Tshibangu T. (2020). [18F]AlF-NOTA-octreotide PET imaging: biodistribution, dosimetry and first comparison with [68Ga]Ga-DOTATATE in neuroendocrine tumour patients. *European Journal of Nuclear Medicine and Molecular Imaging*.

[11] Hou J., Long T., Yang N. (2021). Biodistribution of 18F-AlF-NOTA-octreotide in different organs and characterization of uptake in neuroendocrine neoplasms. *Molecular Imaging and Biology*.

[12] Garin E., Le Jeune F., Devillers A. (2009). Predictive value of18F-FDG PET and somatostatin receptor scintigraphy in patients with metastatic endocrine tumors. *Journal of Nuclear Medicine*.

[13] Binderup T., Knigge U., Loft A., Federspiel B., Kjaer A. (2010). 18F-fluorodeoxyglucose positron emission tomography predicts survival of patients with neuroendocrine tumors. *Clinical Cancer Research*.

[14] Partelli S., Rinzivillo M., Maurizi A. (2015). The role of combined Ga-DOTANOC and (18)FDG PET/CT in the management of patients with pancreatic neuroendocrine tumors. *Neuroendocrinology*.

[15] Nilica B., Waitz D., Stevanovic V. (2016). Direct comparison of (68)Ga-DOTA-TOC and (18)F-FDG PET/CT in the follow-up of patients with neuroendocrine tumour treated with the first full peptide receptor radionuclide therapy cycle. *European Journal of Nuclear Medicine and Molecular Imaging*.

[16] Hofman M. S., Hicks R. J. (2012). Changing paradigms with molecular imaging of neuroendocrine tumors. *Discovery Medicine*.

[17] Chan D. L., Pavlakis N., Schembri G. P. (2017). Dual somatostatin receptor/FDG PET/CT imaging in metastatic neuroendocrine Tumours: proposal for a novel grading scheme with prognostic significance. *Theranostics*.

[18] Laverman P., McBride W. J., Sharkey R. M. (2010). A novel facile method of labeling octreotide with (18)F-fluorine. *Journal of Nuclear Medicine*.

[19] Eisenhauer E. A., Therasse P., Bogaerts J. (2009). New response evaluation criteria in solid tumours: revised RECIST guideline (version 1.1). *European Journal of Cancer*.

[20] Paiella S., Landoni L., Tebaldi S. (2021). Dual-tracer (68Ga-DOTATOC and 18F-FDG-)-PET/CT scan and G1-G2 non-functioning pancreatic neuroendocrine tumors: a single-center retrospective evaluation of 124 non-metastatic resected cases. *Neuroendocrinology*.

[21] Zhang P., Yu J., Li J. (2018). Clinical and prognostic value of PET/CT imaging with combination of (68)Ga-DOTATATE and (18)F-FDG in gastroenteropancreatic neuroendocrine neoplasms. *Contrast Media & Molecular Imaging*.

[22] Soret M., Bacharach S. L., Buvat I. (2007). Partial-volume effect in PET tumor imaging. *Journal of Nuclear Medicine*.

[23] Mehta S., de Reuver P. R., Gill P. (2015). Somatostatin receptor SSTR-2a expression is a stronger predictor for survival than Ki-67 in pancreatic neuroendocrine tumors. *Medicine (Baltimore)*.

[24] Brunner P., Jörg A. C., Glatz K. (2017). The prognostic and predictive value of sstr2-immunohistochemistry and sstr2-targeted imaging in neuroendocrine tumors. *European Journal of Nuclear Medicine and Molecular Imaging*.

[25] Sun Y., Lu P., Yu L. (2016). The volume-metabolic combined parameters from (18)F-FDG PET/CT may help predict the outcomes of cervical carcinoma. *Academic Radiology*.

[26] Hyun S. H., Ahn H. K., Kim H. (2014). Volume-based assessment by (18)F-FDG PET/CT predicts survival in patients with stage III non-small-cell lung cancer. *European Journal of Nuclear Medicine and Molecular Imaging*.

[27] Kim C. Y., Hong C. M., Kim D. H. (2013). Prognostic value of whole-body metabolic tumour volume and total lesion glycolysis measured on ^18^F-FDG PET/CT in patients with extranodal NK/T-cell lymphoma. *European Journal of Nuclear Medicine and Molecular Imaging*.

[28] Altieri B., Di Dato C., Martini C. (2019). Bone metastases in neuroendocrine neoplasms: from pathogenesis to clinical management. *Cancers*.

[29] Rindi G., D'Adda T., Froio E., Fellegara G., Bordi C. (2007). Prognostic factors in gastrointestinal endocrine tumors. *Endocrine Pathology*.

[30] Frilling A., Sotiropoulos G. C., Li J., Kornasiewicz O., Plöckinger U. (2010). Multimodal management of neuroendocrine liver metastases. *HPB: The Official Journal of the International Hepato Pancreato Biliary Association*.

[31] Basu S., Sirohi B., Shrikhande S. V. (2014). Dual tracer imaging approach in assessing tumor biology and heterogeneity in neuroendocrine tumors: its correlation with tumor proliferation index and possible multifaceted implications for personalized clinical management decisions, with focus on PRRT. *European Journal of Nuclear Medicine and Molecular Imaging*.

[32] Kayani I., Conry B. G., Groves A. M. (2009). A comparison of68Ga-DOTATATE and18F-FDG PET/CT in pulmonary neuroendocrine tumors. *Journal of Nuclear Medicine*.

[33] Mapelli P., Partelli S., Salgarello M. (2020). Dual tracer 68Ga-DOTATOC and 18F-FDG PET/computed tomography radiomics in pancreatic neuroendocrine neoplasms: an endearing tool for preoperative risk assessment. *Nuclear Medicine Communications*.

[34] La Rosa S., Inzani F., Vanoli A. (2011). Histologic characterization and improved prognostic evaluation of 209 gastric neuroendocrine neoplasms. *Human Pathology*.

[35] Liu X., Li N., Jiang T. (2020). Comparison of gallium-68 somatostatin receptor and ^18^F-fluorodeoxyglucose positron emission tomography in the diagnosis of neuroendocrine tumours: a systematic review and meta-analysis. *Hellenic Journal of Nuclear Medicine*.

